# Fermentation Characteristics and In Vitro Digestibility of Fibers and Fiber-Rich Byproducts Used for the Feeding of Pigs

**DOI:** 10.3390/ani11020341

**Published:** 2021-01-29

**Authors:** Martin Bachmann, Sebastian Michel, Jörg Michael Greef, Annette Zeyner

**Affiliations:** 1 Institute of Agricultural and Nutritional Sciences, Martin Luther University Halle-Wittenberg, 06120 Halle (Saale), Germany; martin.bachmann@landw.uni-halle.de (M.B.); bas-mi@web.de (S.M.); 2 Institute for Crop and Soil Science, Federal Research Centre for Cultivated Plants, Julius Kühn Institute, 38116 Braunschweig, Germany; joerg-michael.greef@julius-kuehn.de

**Keywords:** dietary fibers, fiber-rich byproducts, physicochemical properties, fermentability, in vitro digestibility

## Abstract

**Simple Summary:**

Inclusion of dietary fibers into the diet may have positive impact on health and wellbeing of pigs. The objective of the study was to examine physicochemical properties of fiber preparations and fiber-rich byproducts in relation to fermentability and digestibility using in vitro batch-culture incubation. Powdered cellulose, *Aspergillus niger* mycelium, which is a byproduct of citric acid production, lucerne chaff, soybean shells, wheat bran, and sugar beet pulp were notably fermented and contributed to the digestible dry matter and organic matter when used as diet constituents. The tested lignocelluloses were not fermented and are rather useful as bulk materials.

**Abstract:**

Dietary fibers may have positive impact on health and wellbeing of pigs. The study examined physicochemical properties of two lignocelluloses (including and excluding bark), powdered cellulose, *Aspergillus niger* mycelium, lucerne chaff, soybean shells, wheat bran, and sugar beet pulp in relation to fermentability and digestibility using in vitro batch-culture incubation. Maize starch and a purified cellulose were used as standardized substrates for classification of the test substrates. The substrates covered a wide range regarding their physicochemical properties. Swelling capacity (SC) was 9–411%, water binding capacity (WBC) was 4.4–14.3 g/g dry matter (DM), and water holding capacity (WHC) was 4.1–10.6 g/g DM. Gas production and other fermentation parameters—namely post-incubation pH, CH_4_, NH_3_, and short chain fatty acids (SCFA) concentrations—revealed a significant fermentation of sugar beet pulp, soybean shells, lucerne chaff, wheat bran, *A. niger* mycelium, and powdered cellulose, whereas the lignocelluloses were not fermented. Significant correlations were found between the physicochemical properties and the fermentation parameters (*p* < 0.05). Enzymatic pre-digestion mostly reduced gas, NH_3_, and SCFA production. In vitro digestibility of DM (IVDMD) and organic matter (IVOMD) was mostly negligible after enzymatic pre-digestion. Fermentation alone led to only 0.10–0.15 IVDMD and 0.14–0.15 IVOMD in lignocelluloses and powdered cellulose, respectively, but 0.44–0.37 IVDMD and 0.46–0.38 IVOMD in the remainder of substrates (*p* < 0.05). In vitro digestibility was again correlated with the physicochemical properties of the substrates and the fermentation parameters (*p* < 0.05). The fiber preparations and fiber-rich byproducts were fermented to a relevant extent. In contrast, lignocelluloses were not fermented and can be used rather as bulk material.

## 1. Introduction

Dietary fibers are important for maintaining health and wellbeing of pigs. High rates of fiber inclusion significantly reduce digestibility of energy, digestibility of organic matter, and digestibility of most of the crude nutrients, and thus reduce the concentration of metabolizable energy in the ration [1,2,3]. This comes mainly from reduced retention time of feeds in the digestive tract and reduced density of digestible nutrients [1,2,3]. However, digestibility of fibers might be increased [4] and stimulation of gut peristalsis also contributes to preventing obstipation, which is relevant especially in pregnant sows [1]. In balanced diets, fibers may have positive dietary effects also in piglets and fattening pigs. Already post-weaning piglets harbor fiber-utilizing microbes, which are part of the intestinal core microbiome [5]. The way and efficiency fiber work largely depend on its swelling capacity (SC), water holding capacity (WHC), level of lignification, and fermentability amongst others [6]. Soluble fiber has high SC and WHC. It thus may contribute to an earlier and more persistent satiety [1]. Sated pigs have less stress and heightened wellbeing [1]. Increasing levels of fiber in the ration stimulate secretion of saliva, gastric juice, and bile, which is stabilizing the gastrointestinal pH [1,7]. Dietary fiber also stimulates cell proliferation and regeneration of the intestinal epithelium [1,8] and helps to maintain a resilient intestinal microflora. Despite of negative effects on the digestibility of many nutrients, inclusion of dietary fiber into the ration therefore may substantially support digestive functions and the health of pigs [1,7,8]. In addition to nutritional effects, fibers may affect the exposition of nitrogenous compounds from solid and liquid manure [3]. As a result of stimulated microbial activity in the large intestine, there is a shift from renal to fecal nitrogen excretion [3,4]. The degradation of microbial protein from feces is slower than nitrogen release from urine [3]. Moreover, volatility of nitrogenous compounds is decreasing in consequence of pH reduction after increase of concentrations of short chain fatty acids (SCFA) from microbial fermentation [3,9].

The objective of the present study was to assess physicochemical properties of commercial fiber preparations and fiber-rich byproducts, and their potential digestibility and hindgut fermentability in weaned piglets. For this, an in vitro batch-culture method with fecal inoculum was used.

## 2. Materials and Methods

### 2.1. Substrates

In this study, the following three fiber preparations were investigated: lignocellulose made from wood (including bark), lignocellulose (excluding bark), and powdered cellulose (CFF GmbH & Co. KG, Ilmenau, Germany). In addition, the following five fiber-rich byproducts were used for comparison: *Aspergillus niger* mycelium as byproduct of citric acid production (CFF GmbH & Co. KG, Ilmenau, Germany), lucerne chaff, soybean shells, wheat bran, and sugar beet pulp. We also added two substrates of laboratory quality, wheat starch (CAS-No. 9005-25-8) and a second powdered cellulose (CAS-No. 9004-34-6) hereafter referred to as purified cellulose. Wheat starch and the purified cellulose are routinely used in our laboratory to classify fermentable substrates in respect of gas production as they mark upper and lower borders of the capacity to be fermented in the batch-culture system. In total, 10 substrates were investigated. The substrates were priorly irradiated with γ-rays at 29.85–34.48 kGy (Synergy Health Radeberg GmbH, Radeberg, Germany) to avoid partial fermentation by adherent microbes. All substrates were pulverized using a Retsch MM 400 ball mill (Retsch GmbH, Haan, Germany).

### 2.2. Enzymatic Pre-Digestion of Substrates

Pre-digestion of the substrates with body-own enzymes was carried out separately for each of seven runs as described by Boisen and Fernández [10] (step 1 and step 2). We used 3.0 g of each substrate. Briefly, 150 mL of 0.1 M phosphate buffer (pH = 6.0) were added. Then, 60 mL of 0.2 M HCl was added. The pH was adjusted to 2.0 using either 1.0 M HCl or 1.0 M NaOH solution, before 6 mL of a porcine pepsin solution (2000 FIP-U/g; 25 mg/mL) and 3 mL of a chloramphenicol solution (0.5 g/100 mL ethanol) were added. The substrates were incubated that way at 39 °C for 2 h. Afterwards, 60 mL of 0.2 M phosphate buffer (pH = 6.8) and 30 mL of 0.6 M NaOH solution were added. The pH was adjusted to 6.8 and 6 mL of porcine pancreatin solution (100 mg/mL) were added. The substrates were incubated at 39 °C for another 4 h. Continuous mixing was ensured by magnetic stirring. Finally, the solutions were filtered through ash-free paper filters (Whatman #41; Cytiva UK Limited, Little Chalfont, UK), washed with distilled water, and dried at 60 °C for 24 h. Starch was decomposed after enzymatic treatment for the most part; it was therefore used without pre-digestion. To maintain comparability, purified cellulose has not been pre-digested as well.

### 2.3. Management and Feeding of Donor Animals

A group of 20 weaned female piglets was available as donors for fecal collection. The piglets were kept, cared for, and used in accordance with animal welfare legislation after approval by the Saxony-Anhalt Animal Welfare Authority (approval no. 2-1527 MLU). The animals were stabled in with five weeks and stabled out with 11 weeks of age. During the time of feces collection, body weight was 15 ± 1.8 kg (week 1), 20 ± 2.5 kg (week 2), 25 ± 3.0 kg (week 3), and 30 ± 2.6 kg (week 4). The piglets were housed in two groups with each 10 animals in running boxes in a stable with induced ventilation and lighting. Within the first and second week post stabling, the piglets received a pre-starter mix ad libitum and 2 mL soybean oil/animal/d. From the third week on, the animals received a starter mix ad libitum. Supplemented soybean oil was 3 mL/animal/d in the third and fourth week, and 4 mL thereafter. Water was offered ad libitum. The pre-starter/starter mixes consisted of wheat (38.00/38.00%), barley (17.73/20.55%), maize (8.00/12.00%), soybean meal (22.00/18.00%), whey powder (5.00/2.50%), wheat bran (3.00/3.00%), soybean oil (2.50/2.40%), monocalcium phosphate (0.50/0.50%), mineral pre-mix (2.00/2.00%), lysine (0.56/0.50%), threonine (0.26/0.22%), methionine (0.22/0.17%), tryptophan (0.06/0.05%), and valine (0.17/0.11%) on as fed basis. The chemical composition of pre-starter and starter compound feed is given in Table 1.

### 2.4. Sampling of Feces and Sample Preparation

Sampling of feces was carried out from the fourth to seventh week post stabling, separately for each in vitro run (usually two times a week). Feces samples were collected by hand without any compulsion or manipulation of the animals and immediately stored at 39 °C. For subsequent preparation of inoculum, feces of minimal 3 and maximal 6 piglets were mixed. Fecal suspension was made by adding 0.9% NaCl-solution at a ratio of 1:5 [11], mixing for 2 min at 230 rpm using a Seward Stomacher^®^ 400 Circulator paddle blender (Seward Ltd., Worthing, UK), and filtering through two layers of cheesecloth. The suspension had a pH of 6.49 ± 0.179 and a redox potential of −279 ± 34.5 mV. The inoculum was prepared by mixing one part of the fecal suspension with two parts of COSITEC buffer [12] under CO_2_ flush. Purging with CO_2_ was continued for 15 min. The buffer contained 0.284 g Na_2_SO_4_, 2.100 g NaHCO_3_, 0.138 g NaH_2_PO_4_ × H_2_O, 0.267 g NH_4_Cl, 0.746 g KCl, 0.368 g CaCl_2_ × 2 H_2_O, 0.508 g MgCl_2_ × 6 H_2_O, and 6.721 g NaCl/L (pH = 7.5) [12]. The final inoculum had a pH of 6.12 ± 0.0622 and a redox potential of −244 ± 43.3 mV.

### 2.5. Batch-Culture Incubation

A total of seven runs was carried out. Each run comprised a single fermenter for the pre-digested and a single fermenter for the not pre-digested version of each of the test substrates, as well as starch, purified cellulose, and blank fermenters (i.e., fecal inoculum without substrate) in duplicate. A quantity of 400 mg of substrate was directly incubated in 60 mL of inoculum in the ANKOM RF Gas Production System (ANKOM Technology, Macedon, NY, USA). The fermenters had an actual volume capacity of 135 ± 2.20 mL (i.e., approximately 75 mL headspace volume). Each bottle was capped with a gas pressure measuring module. The bottles were randomly distributed to three identical shaking water baths at constantly 39 °C with automatic temperature control. The agitation interval was 80 rpm. Prior to incubation, oxygen was purged out of each fermenter by venting with argon through the modules’ Luer ports until the inner pressure exceeded a predefined 8-psi threshold and the valve opened to release the gas. Then, the modules were manually set to a 0-psi threshold to release the entire headspace gas.

### 2.6. Analysis of Gas Production

Cumulative gas pressures were automatically documented by the system every 5 min. For this, a 1.5 psi threshold was set for automatic release of accumulated gases, which prevents supersaturation of fermentation gases in the medium [13]. The valve open time was set to 150 ms. The gas pressures were applied to blank correction using a mean of two blanks per run and converted to mL of gas produced.

### 2.7. Analysis of CH_4_ Production

Gas from the fermenters’ headspace was sampled at three periods throughout incubation (i.e., 2–4, 22–24, and 46–48 h) through the modules’ vent valve using an adapter connected to a gas-proof syringe (SGE Analytical Science, Trajan Scientific and Medical, Ringwood, Australia; 2.5 mL). In the syringe, vacuum was created, the module was activated manually, and gas flowed into the syringe. At least 2 mL gas was collected per sample. The CH_4_ concentration was analyzed by gas chromatography using a Shimadzu GC 2010 Plus (Shimadzu Corp., Kyoto, Japan) fitted with a 250 μL upstream gas loop, a ShinCarbon micropacked column (Restek Corp., Bellefonte, PA, USA; 2 m × 0.53 mm inner diameter, 80/100 mesh size), and a flame ionization detector. Nitrogen was used as carrier gas and makeup gas. The column gas flow was 20.39 mL/min, with 300 kPa pressure at the column. The injection and column temperatures were 45 °C and a split of 13.6 was used. Detection of CH_4_ was performed at 150 °C. The CH_4_ concentrations were expressed as the difference between CH_4_ produced from substrates and CH_4_ produced in blanks using one blank fermenter for each incubation period within each run.

### 2.8. In Vitro Digestibility of Dry Matter and Organic Matter

After enzymatic pre-digestion, the dried filters including the substrates’ residues were weighed, 400 mg of substrate were used for batch-culture incubation, and the rest was dried at 105 °C until constant weight to determine the dry matter (DM) concentration. The dried residues were subsequently ashed to determine the crude ash and organic matter concentrations. After batch-culture incubation, liquid and solid contents of the fermenters were filtered (Whatman #41 filter circles) and washed with distilled water. The filters were subsequently dried at 105 °C to constant weight, cooled off in a desiccator, and weighed before they were ashed, cooled off, and weighed again. Empty filters were used as blanks for correction. The in vitro DM digestibility (IVDMD) and in vitro organic matter digestibility (IVOMD) coefficients were determined according to Noblet and Jaguelin-Peyraud [14].

### 2.9. Additional Analyses and Calculations

Feed analyses were performed using representative bulk samples of the pre-starter and starter mixes fed to the piglets and separate samples of the incubated materials, respectively. All analyses were at least performed in duplicate. Dry matter, crude ash, crude protein (CP), acid ether extract, sugar, crude fiber, and detergent fibers were analyzed according to official methods of the Association of German Agricultural Analytic and Research Institutes (VDLUFA) [15] (methods no. 3.1, 4.1.1, 5.1.1 B, 6.1.1, 6.5.1, 6.5.2, 6.5.3, 7.1.1, and 8.1). Neutral detergent fiber (aNDFom) was determined after treatment with heat-stable amylase, which was added to the neutral detergent solution. Neutral detergent fiber and acid detergent fiber (ADFom) were expressed exclusive of residual ash. Starch was determined using the amyloglucosidase method (VDLUFA method no. 7.2.5) [15]. The gross energy concentration of compound feeds and test substrates was determined by bomb calorimetry using a C7000 Oxygen Bomb Calorimeter (IKA Werke, Staufen, Germany). The concentrations of metabolizable energy in pre-starter and starter were calculated on the basis of nutrient analyses using a multiple regression equation [16]. In the pre-starter and starter compound feeds, proteins were hydrolyzed with hydrochloric acid and amino acids were analyzed according to VDLUFA (method no. 4.11.1) [15] using a Biochrom 30 Amino Acid Analyzer with PEEK-Sodium Prewash Column (100 × 4.6 mm) and PEEK-Oxidized Feedstuff Column (200 × 4.6 mm) (Biochrom Ltd., Cambridge, UK). For the analysis of tryptophan, proteins were hydrolyzed with phosphoric acid and hydrochloric acid. Tryptophan was analyzed following Fontaine et al. [17] by high performance liquid chromatography (Agilent 1100 fitted with ZORBAX Eclipse XDB-C8; 150 × 4.6 mm, 5 μm; Agilent Technologies Inc., Santa Clara, CA, USA). The pH and the redox potential were measured using a Mettler Toledo Seven Excellence unit with InLab^®^ Expert Pro and InLab^®^ Redox electrodes (Mettler Toledo GmbH, Greifensee, Switzerland). The concentration of NH_3_ in the inoculum and fermenter contents was determined using the method of Conway and Byrne [18]. The concentration of organic acids was analyzed after aqueous extraction by gas chromatography. A Shimadzu GC 2010 fitted with flame ionization detector operated at 200 °C and SGE BP21 separation column (30 m × 0.53 mm × 0.5 μm; Trajan Scientific Australia Pty. Ltd., Ringwood, Australia) was used. The injection volume was 0.5 μL at 180 °C on-column. Target analytes were separated with 22.7 kPa constant pressure and the following oven temperature program: 85–200 °C at 8 °C/min, then hold for 6 min. Water binding capacity (WBC) and WHC were determined referring to Kyriazakis and Emmans [19]. In brief, 1 g of substrate was mixed with 20 mL distilled water and was allowed to swell for 24 h. Afterwards, the swollen substrate was either filtered using a Whatman #41 paper filter circle (WBC) or centrifuged for 15 min with 6000× *g* and decanted (WHC). The residues were weighed in wet condition, dried at 105 °C to constant weight, and weighed again to obtain the quantity of water bound or hold. The SC was measured as expansion of volume in a measuring cylinder [20].

### 2.10. Statistical Analysis

Statistical analysis was performed using the SAS 9.4 software package (SAS Institute Inc., Cary, NC, USA). Using boxplots, outliers were identified and removed from the data set. At this, outliers were defined as observations that lay 1.5 interquartile ranges above or below the upper or lower fences of the box. Least squares means were estimated using the MIXED procedure. Substrate alone (including starch and purified cellulose) or substrate, pre-digestion, and their interaction (excluding starch and purified cellulose) were considered as fixed effects. The run was considered as random effect. In part, heterogeneous residual variances were permitted with reference to the substrates or grouped substrates (where group 1 comprised lucerne chaff, soybean shells, wheat bran, and sugar beet pulp, and group 2 comprised powdered cellulose, *A. niger* mycelium, and the lignocelluloses). Heterogeneous variances with reference to the substrates were additionally or alternatively considered for the runs. The studentized residuals were confirmed to have Gaussian distribution by Shapiro–Wilk and/or Kolmogorov–Smirnov test. Non-linear regression analysis was performed upon gas production kinetics over the 48 h incubation periods using the MODEL procedure and Gompertz function [21]. Pearson correlation coefficients between and among the substrates’ physicochemical properties and fermentation parameters were computed using the CORR procedure. For statistical tests, the level of significance was set to *p* < 0.05.

## 3. Results

### 3.1. Chemical Composition and Physicochemical Properties of Test Substrates

The chemical composition of test substrates is given in Table 2. The fiber preparations were characterized by large concentrations of detergent fibers and gross energy. Powdered cellulose was significantly reduced in gross energy compared to the other fiber preparations. *A. niger* mycelium had a remarkable CP content (144 g/kg DM). The other selected byproducts were rich in detergent fibers and also had considerable CP concentrations. Wheat bran additionally had a noteworthy concentration of starch (134 g/kg DM).

The physicochemical properties of the test substrates are summarized in Table 2. Swelling capacity, WBC, and WHC were widely spread among the analyzed substrates.

### 3.2. Total Gas and CH_4_ Production

**Figure 1:**
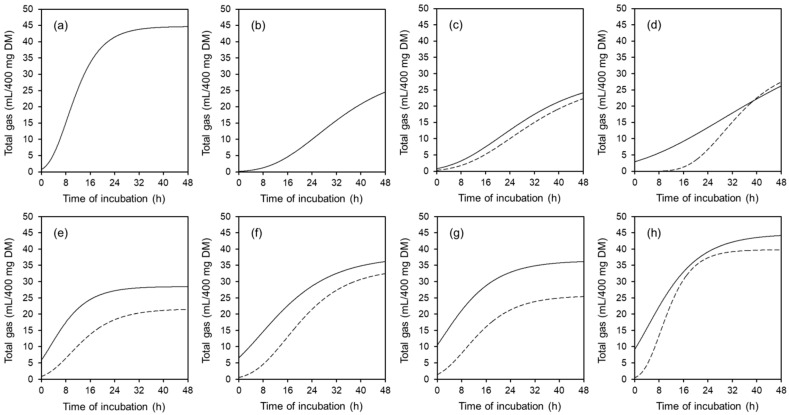
Cumulative gas production of starch (**a**), purified cellulose (**b**), powdered cellulose (**c**), mycelium of *Aspergillus niger* (**d**), lucerne chaff (**e**), soybean shells (**f**), wheat bran (**g**), and sugar beet pulp (**h**), with pre-digestion (dashed line) and without pre-digestion (solid line), modeled by Gompertz non-linear regression function. DM: dry matter.

**Figure 2:**
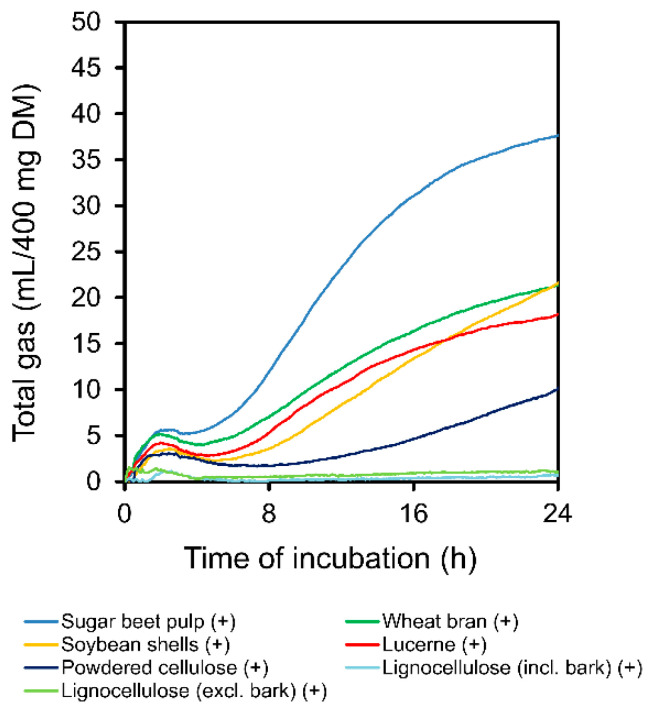
Raw data plot of mean gas production during 24 h of incubation of fibers and fiber-rich feeds, which had been pre-digested (+). DM: dry matter.

Gas production after 36 (GP_36_) and 48 h of incubation (GP_48_) is shown in Table 3 for materials that have not been pre-digested. The GP_36_ and GP_48_ differed markedly among the substrates (*p* < 0.05). Starch and sugar beet pulp had highest GP_36_ and GP_48_, the lignocelluloses were by contrast barely fermented. Inclusion or exclusion of bark did not affect gas production from lignocellulose (*p* > 0.05). Among the tested fiber preparations, *A. niger* mycelium had with 20.4 (GP_36_) and 23.1 mL/400 mg DM (GP_48_) the highest gas production. Gas production was reduced after enzymatic pre-digestion with lucerne chaff, soybean shells, and wheat bran (*p* < 0.01), as well as with sugar beet pulp and the lignocelluloses (*p* > 0.05; Table 3). With *A. niger* mycelium, GP_48_ slightly increased after pre-digestion (*p* > 0.05). Except for sugar beet pulp, gas production increased from hour 36 to 48 of incubation. The differences in gas production among the substrates and the effect of enzymatic pre-digestion were likewise reflected by the progression of gas production (Figure 1) and the resulting model estimates (Table 4). Incubation of lignocelluloses resulted in marginal gas production with a widely not convergent shape. Therefore, curve fitting was not applied in lignocelluloses. In the pre-digested materials, a biphasic shape of gas production was visible with a first peak around 2 h and a second gradual increase starting at 6 h after incubation (Figure 2).

The concentration of CH_4_ in the fermentation gas increased with progressing time of incubation. Significant quantities of CH_4_ were produced still from blank fermentation (53 ± 22 µmol/L at 2–4 h, 506 ± 172 µmol/L at 22–24 h, and 769 ± 197 µmol/L at 46–48 h of the incubation period). The CH_4_ concentrations differed among the substrates (*p* < 0.05; Table 5). Lucerne chaff, soybean shells, and wheat bran showed high CH_4_ production from the beginning on. Highest CH_4_ production at late incubation times (46–48 h) was with the cellulose preparations. The consistently lowest CH_4_ production was with the lignocelluloses, which had maximal 743 μmol/L including bark and 324 μmol/L excluding bark (*p* > 0.05). The *A. niger* mycelium showed moderate CH_4_ production (maximal 1105 μmol/L). Enzymatic pre-digestion led to lower CH_4_ concentration in the fermentation gas until 24 h of incubation (Table 5). This was significant in *A. niger* mycelium, lucerne chaff, soybean shells, wheat bran, and sugar beet pulp (*p* < 0.05).

### 3.3. Post-Incubation pH, NH_3_, and Short Chain Fatty Acids

The pH-values measured in fecal slurry after fermentation of the substrates are summarized in Table 6. The pre-incubation pH was 6.12 for all fermenters. In the blanks, pH slightly increased throughout the incubation period. There was no pH reduction with the lignocelluloses. Fermentation of the other substrates led to specifically graduated pH reduction, which was most pronounced in sugar beet pulp and starch. The post-incubation pH differed among the substrates (*p* < 0.05).

Pre-incubation NH_3_ concentration in fecal suspension was 4.49 ± 0.559 mmol/L. A part of NH_3_ occurred still from the inoculum (9.75 ± 1.71 mmol/L in blanks). Based on that, no NH_3_ came from fermentation of starch, sugar beet pulp, or lignocelluloses (Table 7). Ammonia concentrations increased during fermentation of *A. niger* mycelium, soybean shells, lucerne chaff, and wheat bran. They were regularly lower after enzymatic pre-digestion, especially in *A. niger* mycelium, lucerne chaff, wheat bran, and sugar beet pulp (*p* < 0.05; Table 7).

Prior to incubation, the fecal suspension had 3.89 ± 0.932 mmol acetic acid/L, 1.90 ± 0.165 mmol propionic acid/L, 0.31 ± 0.026 mmol *i*-butyric acid/L, 1.06 ± 0.188 mmol *n*-butyric acid/L, 0.34 ± 0.050 mmol *i*-valeric acid/L, 0.36 ± 0.041 mmol *n*-valeric acid/L, and 0.08 ± 0.02 mmol *n*-caproic acid/L. After fermentation, blank fermenters had 6.91 ± 1.656 mmol acetic acid/L, 3.10 ± 0.449 mmol propionic acid/L, 0.50 ± 0.098 mmol *i*-butyric acid/L, 1.38 ± 0.520 mmol *n*-butyric acid/L, 0.72 ± 0.15 mmol *i*-valeric acid/L, 0.63 ± 0.13 mmol *n*-valeric acid/L, and 0.18 ± 0.029 mmol *n*-caproic acid/L. Post-incubation concentrations of SCFA in fecal slurry are given in Table 8. Production of *i*-butyric acid, *i*-valeric acid, and *n*-caproic acid did not occur from fermentation of the substrates; the concentrations were lower than 0.20 mmol/L. It was mainly acetic acid and propionic acid that was produced during fermentation of the substrates (Table 8). The proportion of SCFA concentrations (C_2_ acetic acid: (C_3_ propionic acid + C_4_ butyric acid)) was between 0.8 (starch) and 3.1 (powdered cellulose) excluding the lignocelluloses. Only acetic acid was marginally produced from fermentation of lignocelluloses. Significant production of SCFA was found in sugar beet pulp, soybean shells, lucerne chaff, wheat bran, *A. niger* mycelium, and the cellulose preparations (Table 8). Fatty acid concentrations differed among the substrates (*p* < 0.05). Enzymatic pre-digestion mostly reduced the production of SCFA from the substrates (*p* > 0.05; Table 8).

### 3.4. In Vitro Digestibility of Dry Matter and Organic Matter

In vitro DM digestibility after enzymatic pre-digestion differed among the tested substrates (*p* < 0.05; Table 9). The IVDMD was 0.06 in *A. niger* mycelium and lower than 0.06 in the other fiber preparations. Among fiber-rich byproducts, IVDMD ranged between 0.04 (soybean shells) and 0.37 (wheat bran). Batch-culture fermentation without pre-digestion led to higher IVDMD in wheat bran, sugar beet pulp, soybean shells, *A. niger* mycelium, and lucerne chaff than in lignocelluloses and powdered cellulose (*p* < 0.05). Total IVDMD again differed among the substrates (*p* < 0.05). It was highest in sugar beet pulp and lowest in lignocellulose.

The IVOMD differed among the substrates after pre-digestion (*p* < 0.05), but was consistently 0.01 or lower (Table 10). After fermentation without pre-digestion, IVOMD remained on a negligible level (maximal 0.04). Total IVOMD also differed among the substrates (*p* < 0.05). Inclusion or exclusion of bark in preparation of lignocellulose affected IVDMD or IVOMD on no account (*p* > 0.05).

### 3.5. Correlations

Pearson correlation coefficients between and among the physicochemical properties of the tested substrates and the fermentation parameters are summarized in Table 11. Significant correlation was found between WBC and WHC (*r* = 0.85; *p* < 0.05) and to some extent between WBC and SC (*r* = −0.27; *p* < 0.05). Notable correlation has also been found between WHC and SC and pH (*r* = −0.43 and *r* = −0.68, respectively; *p* < 0.05), gas production (*r* = 0.48–0.52; *p* < 0.05), and production of SCFA (C_2_, C_3_, or C_4_; *r* = 0.33–0.64; *p* < 0.05). Swelling capacity was correlated with IVDMD (*r* = 0.45; *p* < 0.05) and IVOMD (*r* = 0.48; *p* < 0.05). Strong correlation was among gas production, production of SCFA, pH reduction, and in vitro digestibility (*p* < 0.05; Table 11).

## 4. Discussion

Inclusion of commercial fiber preparations or fiber-rich byproducts into pig diets may impair digestibility of energy and organic matter, and, consequently, the performance of the animals [1,22]. Pigs may likewise benefit from dietary fiber supplementation, which mainly refers to gut health and digestive function, satiety, and wellbeing [1,7,8,22]. The way in which fiber preparations contribute to nutrition and health maintenance depends on fiber source, type, and inclusion level [22]. Having sufficient knowledge on nutrient composition and density, physicochemical properties, potential fermentability, and digestibility of the preparations or byproducts is therefore an application prerequisite. The objective of this study was to assess physicochemical properties of a selection of commercial fiber preparations and fiber-rich byproducts, and their potential digestibility and hindgut fermentability in vitro.

### 4.1. Chemical Composition and Physicochemical Properties

The tested fiber preparations and fiber-rich byproducts were naturally high in detergent fibers and gross energy. The latter are drastically modified in nutrient composition compared with their starting product. Significant residuals of CP, starch, and sugar were partly present in the substrates; however, they are rather no relevant nutrient suppliers on the scale of the entire ration. The mycelial cell walls of *A. niger* are mainly composed of neutral carbohydrates (glucose, mannose, arabinose, glucosamine, and galactosamine), hexosamines, and amino acids [23]. Their composition differs from that of plant fibers, but in the current analysis, they were recorded as detergent fibers as well.

The physicochemical properties of the substrates may significantly affect fermentability. The analyzed WBC, WHC, and SC values were generally within the range reported by previous studies [19,20], although the used analytical methods may have differed in parts. The SC of sugar beet pulp and lignocelluloses was considerably lower than given by Slama et al. [20]. The physicochemical properties of a substrate mainly depend on its (fiber) composition and structure. Large WHC, as found e.g., in powdered cellulose, is associated with feeds that have low bulk density [24]. Such feeds could have gas pockets within their cell wall matrix, which retain water when it is in excess [24]. That way, they are a source of moisture for the growth and metabolic activities of microorganisms [25]. In the digestive tract, they can have a low transit rate, which supports efficient digestion and nutrient absorption [24]. Also, substrates that have a high SC, such the *A. niger* mycelium, increase the surface area and promote microbial colonization and degradation [26].

### 4.2. Fermentation Characteristics

The gas production from fermentation of the substrates in the batch-culture system was analyzed after 36 and 48 h of incubation and for the entire progression within 48 h. The specifically selected incubation times indicate typical solid-phase mean retention times associated with fibers or feeds or diets rich in fiber within the large intestine or total digestive tract of pigs [2,27]. Gas production was in absolute terms lower than measured previously using wheat bran (63 vs. 124–180 mL/g DM) or sugar beet pulp (98 vs. 211–280 mL/g DM) as example [28,29,30,31]. This is a result of inconsistency of in vitro methods and individual sources of variation [32]. Independent of this, a reliable ranking of the fermentability of different substrates is possible. Pure starch and sugar beet pulp had highest gas production capacity, because they have high percentages of readily soluble carbohydrates that are easily available for microbial fermentation. By contrast, lignocelluloses were fermented little or not at all. The tested cellulose preparations were chemically and mechanically purified, which led to significantly reduced or completely broken-down parts of lignin (e.g., 1 g acid detergent lignin, ADL/kg DM in powdered cellulose). By contrast, lignocelluloses had 315 and 246 g ADL/kg DM including or excluding bark, respectively, which makes them more recalcitrant towards microbial attack [33,34]. Phenolic acids (e.g., ferulic acid and *p*-coumaric acid) and their esters (arabinoxylans) present in plant cell walls further limit potential biodegradation [35]. This led to considerable differences in fermentability and resulting gas production. The *A. niger* mycelium was well fermented in the batch-cultures. As mentioned above, it had a high SC (367 g/g DM), which might have supported fermentation. Pre-digestion with body-own enzymes reduced subsequent gas production in the majority of substrates, because readily fermentable components such as soluble starch, sugars, or nitrogen compounds disappeared [29]. The measured progressions of gas production were typical for the substrates and widely similar to such illustrated by previous studies [28,29,36]. Enzymatic pre-digestion resulted in a bi-phasic shape of gas production. Easier fermentable components were rapidly degraded followed by slow fermentation of more closely bound parts of the substrates after a certain time delay. Not pre-digested substrates had a larger proportion of easily fermentable components. The longer-lasting fermentation of this portion probably covered the bi-phasic process.

Microbial hydrolysis of cell wall polymers also results in the production of SCFA, H_2_, and CO_2_ [37], followed by pH reduction. The majority of H_2_ is thereby produced synergistically with acetate [38]. In the hindgut of pigs, acetogenic and methanogenic microbes coexist [39]. However, methanogenesis clearly represents the main terminal electron sink reaction [39]. In the batch-cultures, SCFA accumulate with progressing incubation time. Next, there is an increasing need to recover H_2_, which is why CH_4_ increased even in the absence of added substrate.

In lucerne chaff, soybean shells, and wheat bran, CH_4_ production was high from the beginning on. The highest CH_4_ production at late incubation times (46–48 h) was with the cellulose preparations. Lowest CH_4_ production was with the lignocelluloses, because they were not fermented to a relevant extent. Enzymatic pre-digestion led to lower CH_4_ concentration in the fermentation gas until 24 h of incubation, but not at a later stage. This was distinct in *A. niger* mycelium, lucerne chaff, soybean shells, wheat bran, and sugar beet pulp, substrates that have a significant proportion of easily fermentable components.

Post-incubation pH reduction was logically graduated following fermentation intensity. It was most distinct with starch and sugar beet pulp. The pH was not reduced with the lignocelluloses.

Substrates with a larger proportion of fermentable components promote microbial growth and microbial nitrogen uptake for growth is higher than with pure fibers [30]. Substrates with relevant CP concentration may provide more nitrogen as can be incorporated by the microorganisms; then, NH_3_ concentration is increasing in the inoculum. The concentration of NH_3_ was highest with the *A. niger* mycelium, soybean shells, lucerne chaff, and wheat bran, in contrast to the negligible NH_3_ concentration found with starch, sugar beet pulp, celluloses, and lignocelluloses. This confirms previous in vitro observations [40,41]. Hence, enzymatic pre-digestion reduced NH_3_ emissions.

The molar percentage of SCFA in the inoculum depends on the composition of the substrates, their rate of degradation, and digestibility [37,42]. In general, the substrates with the more accessible and digestible carbohydrate fractions forced the production of propionate and butyrate (e.g., the C_2_:(C_3_+C_4_) ratio was 1.3 with wheat bran, 1.5 with *A. niger* mycelium, or 1.8 with sugar beet pulp), whereas acetate, and as a result H_2_ and CH_4_, were primarily produced from pure fibers (e.g., the C_2_:(C_3_+C_4_) ratio was 3.1 with powdered cellulose). This confirms previous observations made by Stagonias and Pearce [43] or Carneiro et al. [44]. Increased propionate and especially increased butyrate production can be advantageous, because they are relevant sources of energy for epithelial cells and modulate immune and inflammatory responses in the intestine [45,46,47,48,49]. At this, the SCFA are utilized via β-oxidation to acetyl-CoA and the citric acid cycle [50]. In lignocelluloses, just a marginal production of acetic acid occurred, which was somewhat higher in the presence of bark (0.85 vs. 1.45 mmol/L). The production of SCFA was higher with not pre-digested substrates. The C_2_:(C_3_+C_4_) ratio, however, was not markedly affected by pre-digestion (e.g., with wheat bran, *A. niger* mycelium, sugar beet pulp, or powdered cellulose it increased or decreased by maximal 0.2 points).

### 4.3. In Vitro Digestibility of Dry Matter and Organic Matter

The tested fiber preparations and fiber-rich byproducts have not been degraded by digestive enzymes. In lucerne chaff, wheat bran, and sugar beet pulp, IVDMD was up to 37% after pre-digestion, but IVOMD was less than 4%. Soluble inorganic compounds were likely washed away with the buffer solution and thus disappeared from the filter residues. This apparently reduced IVOMD. However, also low molecular compounds from organic matter degradation could have been lost that way, which might have not been recognized by IVOMD. The intensity of fermentation is clearly linked to digestibility [42]. The graduation of substrate fermentation was widely reproduced by IVDMD and IVOMD. At this, sugar beet pulp, soybean shells, and wheat bran had the highest IVDMD and IVOMD, although they were lower compared to previous studies [28,29,30,51]. The *A. niger* mycelium and powdered cellulose had 28 and 14% IVDMD and 31 and 21% IVOMD, which correlates to nutrient density. In case of lignocelluloses, only the bark might have been digested.

### 4.4. Correlations

Significant correlation existed between WHC and gas production, SCFA production, and pH reduction, respectively. As expected, strong correlation was also found among gas production, production of SCFA, pH reduction, and in vitro digestibility of not pre-digested substrates. The SC and WHC were closely correlated with the fermentation parameters as they affect microbial colonization [24,26]. Both CH_4_ production and NH_3_ concentration did not clearly correlate with the substrates’ physicochemical properties or with the other fermentation parameters.

## 5. Conclusions

The objective of this study was to assess physicochemical properties of selected commercial fiber preparations and fiber-rich byproducts, and their digestibility and hindgut fermentability in weaned piglets using a fecal batch-culture model. In conclusion, in vitro results on total gas and CH_4_ production, post-incubation pH, as well as NH_3_ and SCFA concentrations in the batch-culture fermenters have shown that the tested fiber preparations and byproducts were fermented to a relevant extent, graduated based on their chemical composition and physicochemical properties, considering usual retention times within the gastrointestinal tract of pigs. The lignocelluloses, however, were not notably fermented. The fiber preparations and byproducts widely repelled pre-digestion by body-own enzymes, as they only contained a few readily soluble components. In vitro DM digestibility and IVOMD of 37–44% and 38–46%, respectively, confirmed a specific hindgut fermentation. By contrast, IVDMD and IVOMD of lignocelluloses were maximal 15%.

Despite the fact that in vitro tests can only partially reflect natural digestive processes, the results confirm the use of lignocelluloses as bulk material to increase crude fiber concentration of pig diets. This might have positive effects on satiety and gut health. The other substrates, especially powdered cellulose and *A. niger* mycelium, may also contribute to the production of SCFA in the hindgut of pigs. Prospectively, in vivo studies are required to countercheck the presented results and to better assess the possible applications of the substrates.

## Figures and Tables

**Table 1:** Chemical composition of the pigs’ feeds offered during the experiment ^1^.

Item	Pre-Starter	Starter
Dry matter	911	907
Crude ash	53	46
Crude protein	241	228
Lys	15.9	13.9
Met	5.8	5.2
Cys	3.9	3.6
Thr	11.5	10.3
Trp	3.7	3.3
Val	12.7	11.3
AEE	48	48
Crude fiber	38	39
aNDFom	217	220
ADFom	54	48
ADL	9	11
Sugar	58	38
Starch	411	438
Gross energy	19.2	19.1
ME_S_	15.7	15.7

ADFom: acid detergent fiber (expressed exclusive of residual ash); ADL: acid detergent lignin; AEE: acid ether extract; aNDFom: neutral detergent fiber (assayed with amylase and expressed exclusive of residual ash); Cys: cysteine; Lys: lysine; Met: methionine; ME_S_: metabolizable energy for pigs; Thr: threonine; Trp: tryptophan; Val: valine. Dry matter (DM) is given as g/kg, gross energy and ME_S_ are given as MJ/kg DM, and all other analytes are given as g/kg DM. ^1^ Analyzed from a single representative bulk sample each.

**Table 2:** Chemical composition and physicochemical properties of test substrates ^1^.

Substrate	DM	CA	CP	AEE	CF	aNDFom	ADFom	ADL	Sugar	Starch	GE	WBC	WHC	SC
Lignocellulose (including bark)	933	13	11	1	679	897	779	315	4	n.a.	20.4	6.7	5.4	80
Lignocellulose (excluding bark)	938	2	5	2	701	918	763	246	15	n.a.	20.1	8.2	6.2	44
Powdered cellulose	939	2	7	4	773	999	966	1	0	n.a.	17.4	14.3	9.7	9
*A. niger* mycelium ^2^	904	3	144	30	373	857	602	221	3	n.a.	19.6	4.4	4.1	367
Lucerne chaff	936	73	151	18	313	474	374	82	29	21	18.8	7.7	8.0	110
Soybean shells	912	51	112	15	414	707	508	18	16	3	17.7	7.5	6.8	240
Wheat bran	919	58	163	47	125	508	156	43	59	134	19.5	6.4	6.4	59
Sugar beet pulp	921	68	105	6	188	500	235	25	61	6	17.3	10.4	10.6	411

ADFom: acid detergent fiber (expressed exclusive of residual ash); ADL: acid detergent lignin; AEE: acid ether extract; aNDFom: neutral detergent fiber (assayed with amylase and expressed exclusive of residual ash); CA: crude ash; CF: crude fiber; CP: crude protein; DM: dry matter; GE: gross energy; n.a.: not analyzed; SC: swelling capacity; WBC: water binding capacity; WHC: water holding capacity. DM is given as g/kg, GE is given as MJ/kg DM, WBC and WHC are given as g water/g DM, SC is measured as expansion in volume (%), and all other analytes are given as g/kg DM. ^1^ Analyzed from a single representative sample. ^2^ Note that in the current analysis the mycelial structural carbohydrates were recorded as detergent fibers but are not to be equated with plant fibers.

**Table 3:** Least squares means of gas production (mL/400 mg dry matter) after 36 (GP_36_) and 48 h of incubation (GP_48_) and the effect of enzymatic pre-digestion of the substrates.

Substrate	GP_36_	GP_48_	GP_36_		GP_48_	
			−	+	−	+
Starch	44.6 ^a^	45.9 ^a^				
Purified cellulose	18.7 ^cde^	23.9 ^cd^				
Lignocellulose (including bark)	5.3 ^ef^	6.4 ^ef^	5.2	1.5	6.6	1.7
Lignocellulose (excluding bark)	1.9 ^f^	2.7 ^f^	2.5	1.8	2.5	2.0
Powdered cellulose	17.8 ^de^	22.2 ^de^	18.0	17.5	22.3	22.4
*A. niger* mycelium	20.4 ^cde^	23.1 ^cd^	20.5	19.4	24.2	27.9
Lucerne chaff	28.4 ^bcd^	28.9 ^bcd^	28.0 ^A^	20.7 ^B^	28.9 ^A^	21.6 ^B^
Soybean shells	34.0 ^bc^	35.1 ^b^	33.0 ^A^	28.9 ^B^	35.3 ^A^	32.7 ^B^
Wheat bran	34.2 ^b^	36.6 ^b^	34.4 ^A^	24.5 ^B^	35.4 ^A^	25.3 ^B^
Sugar beet pulp	43.5 ^ab^	43.5 ^ab^	42.8	39.4	42.6	39.3

Standard errors ranged from 0.301 to 4.56 mL/400 mg dry matter. ^a–f^ Within a column, different superscripts indicate difference among not pre-digested substrates (*p* < 0.05). ^A,B^ Within a row, different upper-case superscripts indicate difference between not pre-digested (−) and pre-digested (+) materials (*p* < 0.01).

**Table 4:** Estimates of model parameters of gas production courses.

Substrate	Pre-Digestion	*a*	*b*	*b + c*	*R* ^2^
Starch	−	44.7	8.5	14.5	0.993
Purified cellulose	−	31.4	26.1	41.7	0.996
Powdered cellulose	−	29.3	21.1	37.6	0.991
	+	28.2	24.5	40.8	0.990
*A. niger* mycelium	−	44.0	28.9	57.9	0.922
	+	33.1	29.3	40.5	0.998
Lucerne chaff	−	28.5	3.1	9.9	0.984
	+	21.6	9.5	17.7	0.991
Soybean shells	−	37.8	7.3	20.4	0.993
	+	34.2	15.6	26.7	0.997
Wheat bran	−	36.4	2.1	11.7	0.971
	+	25.7	9.3	18.1	0.993
Sugar beet pulp	−	44.6	4.5	14.1	0.984
	+	39.8	8.5	14.1	0.995

*a*: asymptotic maximal gas production (mL/400 mg dry matter); *b*: time (h) until which one-third of *a* is produced; *b + c*: time (h) until approximately 70% of *a* is produced.

**Table 5:** Least squares means of CH_4_ concentration (μmol/L) in fecal inoculum expressed as the difference between CH_4_ produced from substrates and CH_4_ produced in blanks at three different periods during 48 h of incubation and the effect of enzymatic pre-digestion of the substrates.

Substrate	2–4 h	22–24 h	46–48 h	2–4 h		22–24 h		46–48 h	
				−	+	−	+	−	+
Starch	70 ^e^	1257 ^b^	1338 ^c^						
Purified cellulose	43 ^f^	846 ^c^	2387 ^a^						
Lignocellulose (including bark)	15 ^fg^	−11 ^e^	743 ^de^	12	7	−28	3	681	671
Lignocellulose (excluding bark)	4 ^g^	−26 ^e^	324 ^e^	6 ^A^	−11 ^B^	10	−100	459	1023
Powdered cellulose	33 ^f^	881 ^c^	2251 ^a^	33	27	903	755	2225	2182
*A. niger* mycelium	59 ^e^	417 ^d^	1105 ^cd^	57 ^A^	−2 ^B^	411 ^A^	244 ^B^	1164	1336
Lucerne chaff	206 ^d^	1542 ^a^	2240 ^a^	240 ^A^	65 ^B^	1539 ^A^	873 ^B^	2206 ^A^	1530 ^B^
Soybean shells	239 ^c^	1366 ^ab^	2236 ^a^	239 ^A^	61 ^B^	1359 ^A^	995 ^B^	2220	2136
Wheat bran	388 ^a^	1264 ^b^	1899 ^b^	380 ^A^	70 ^B^	1257 ^A^	713 ^B^	1882	1531
Sugar beet pulp	316 ^b^	1140 ^b^	1761 ^bc^	315 ^A^	140 ^B^	1141 ^B^	1576 ^A^	1755	1945

Standard errors ranged from 3.692 to 654.9 μmol/L. ^a–g^ Within a column, different superscripts indicate difference among the not pre-digested substrates (*p* < 0.05). ^A,B^ Within a row, different upper-case superscripts indicate difference between not pre-digested (−) and pre-digested (+) materials (*p* < 0.05).

**Table 6:** Least squares means of post-incubation pH-values ^1^.

Substrate ^2^	pH
Blank (only fecal inoculum)	6.44 ^a^
Starch	4.75 ^f^
Purified cellulose	5.67 ^bcd^
Lignocellulose (including bark)	6.39 ^a^
Lignocellulose (excluding bark)	6.48 ^a^
Powdered cellulose	5.80 ^bc^
*A. niger* mycelium	5.43 ^d^
Lucerne chaff	5.84 ^b^
Soybean shells	5.52 ^d^
Wheat bran	5.67 ^c^
Sugar beet pulp	5.12 ^e^

Standard errors ranged from 0.0261 to 0.109. ^a–f^ Different superscripts indicate difference among the substrates (*p* < 0.05). ^1^ A standardized inoculum with an initial mean pH of 6.12 ± 0.0622 was used for all substrates (specifications are given in the text). ^2^ Not pre-digested.

**Table 7:** Least squares means of NH_3_ concentration (mmol/L) in fecal inoculum expressed as the difference between NH_3_ concentration from fermentation of substrates and NH_3_ concentration from background fermentation in blanks and the effect of enzymatic pre-digestion of the substrates.

Substrate	NH_3_	NH_3_	
		−	+
Starch	−4.96 ^f^		
Purified cellulose	0.54 ^d^		
Lignocellulose (including bark)	−0.14 ^d^	−0.14	0.38
Lignocellulose (excluding bark)	−0.17 ^d^	−0.17	−0.19
Powdered cellulose	0.59 ^d^	0.59	0.45
*A. niger* mycelium	1.38 ^d^	1.38 ^A^	0.38 ^B^
Lucerne chaff	3.57 ^b^	3.57 ^A^	1.43 ^B^
Soybean shells	2.40 ^c^	2.40	1.63
Wheat bran	6.75 ^a^	6.75 ^A^	2.55 ^B^
Sugar beet pulp	−1.79 ^e^	−1.79 ^B^	0.22 ^A^

Standard errors ranged from 0.452 to 0.491 mmol/L. ^a–f^ Within a column, different superscripts indicate difference among the not pre-digested substrates (*p* < 0.05). ^A,B^ Within a row, different upper-case superscripts indicate difference between not pre-digested (−) and pre-digested (+) materials (*p* < 0.05).

**Table 8:** Least squares means of concentrations of short chain fatty acids (mmol/L) in fecal inoculum expressed as the difference between fatty acid produced from substrates and fatty acid produced in blanks and the effect of enzymatic pre-digestion of the substrates.

Substrate	Acetic Acid	Propionic Acid	*n*-Butyric Acid	*n*-Valeric Acid	Acetic Acid		Propionic Acid		*n*-Butyric Acid		*n*-Valeric Acid	
					−	+	−	+	−	+	−	+
Starch	9.78 ^bc^	9.71 ^a^	3.14 ^a^	1.01 ^a^								
Purified cellulose	7.70 ^cd^	2.21 ^d^	0.50 ^c^	0.03 ^e^								
Lignocellulose (including bark)	1.45 ^e^	0.02 ^e^	0.24 ^cd^	−0.02 ^ef^	1.45	0.10	0.04	−0.26	0.24	−0.22	−0.02	−0.07
Lignocellulose (excluding bark)	0.85 ^e^	−0.11 ^e^	−0.19 ^d^	−0.08 ^f^	0.85	−0.06	−0.11	−0.40	−0.19	−0.27	−0.08	−0.08
Powdered cellulose	6.57 ^d^	1.82 ^d^	0.27 ^c^	−0.04 ^ef^	6.57	6.41	1.82	1.68	0.27	0.35	−0.04	−0.04
*A. niger* mycelium	6.83 ^d^	3.75 ^cd^	0.94 ^bc^	0.13 ^de^	6.83	6.58	3.75	3.53	0.94	1.31	0.13	0.22
Lucerne chaff	10.18 ^abc^	2.83 ^d^	1.18 ^b^	0.31 ^cd^	10.18	7.54	2.83	1.80	1.18 ^A^	0.37 ^B^	0.31 ^A^	0.04 ^B^
Soybean shells	13.21 ^a^	4.15 ^c^	1.44 ^b^	0.23 ^d^	13.21	10.84	4.15	3.36	1.44	0.97	0.23	0.12
Wheat bran	9.59 ^c^	4.54 ^c^	2.86 ^a^	0.62 ^b^	9.59	6.72	4.54	2.86	2.86	1.88	0.63 ^A^	0.13 ^B^
Sugar beet pulp	13.27 ^ab^	6.16 ^b^	1.25 ^b^	0.45 ^c^	13.27	12.70	6.16	5.75	1.25	2.14	0.45	0.35

Standard errors ranged between 0.0144 and 1.40 mmol/L. ^a–f^ Within a column, different superscripts indicate difference among the not pre-digested substrates (*p* < 0.05). ^A,B^ Within a row, different upper-case superscripts indicate difference between not pre-digested (−) and pre-digested (+) materials (*p* < 0.05).

**Table 9:** Least squares means of in vitro dry matter digestibility coefficients after enzymatic pre-digestion, batch-culture fermentation (without pre-digestion), and in total (after fermentation of pre-digested material).

Substrate	Pre-Digestion	Fermentation	Total
Lignocellulose (including bark)	0.02 ^DE^	0.15 ^B^	0.07 ^E^
Lignocellulose (excluding bark)	0.03 ^D^	0.12 ^B^	0.09 ^DE^
Powdered cellulose	<0.01 ^E^	0.10 ^B^	0.14 ^CD^
*A. niger* mycelium	0.06 ^C^	0.40 ^A^	0.28 ^B^
Lucerne chaff	0.19 ^B^	0.37 ^aA^	0.22 ^bBCD^
Soybean shells	0.04 ^CDE^	0.40 ^A^	0.35 ^B^
Wheat bran	0.37 ^A^	0.44 ^aA^	0.25 ^bBC^
Sugar beet pulp	0.22 ^B^	0.40 ^bA^	0.55 ^aA^

Standard errors ranged from 0.014 to 0.057. ^a,b^ Different superscripts indicate difference between not pre-digested and pre-digested materials (*p* < 0.05). ^A–E^ Within a column, different upper-case superscripts indicate difference among the substrates (*p* < 0.05).

**Table 10:** Least squares means of in vitro organic matter digestibility coefficients after enzymatic pre-digestion, batch-culture fermentation (without pre-digestion), and in total (after fermentation of pre-digested material).

Substrate	Pre-Digestion	Fermentation	Total
Lignocellulose (including bark)	<0.01 ^E^	0.15 ^B^	0.07 ^E^
Lignocellulose (excluding bark)	0.01 ^DE^	0.14 ^B^	0.11 ^DE^
Powdered cellulose	0.01 ^CDE^	0.14 ^B^	0.21 ^BCD^
*A. niger* mycelium	0.01 ^B^	0.40 ^A^	0.31 ^BC^
Lucerne chaff	0.02 ^ABCDE^	0.38 ^aA^	0.21 ^bBC^
Soybean shells	0.03 ^ABCD^	0.42 ^A^	0.34 ^B^
Wheat bran	0	0.46 ^aA^	0.21 ^bC^
Sugar beet pulp	0.04 ^A^	0.45 ^bA^	0.60 ^aA^

Standard errors ranged from 0.002 to 0.054. ^a,b^ Different superscripts indicate difference between not pre-digested and pre-digested materials (*p* < 0.05). ^A–E^ Within a column, different upper-case superscripts indicate difference among the substrates (*p* < 0.05).

**Table 11:** Pearson correlation coefficients between and among physicochemical properties of not pre-digested test substrates and fermentation parameters.

	WBC	WHC	SC	pH	GP_36_	GP_48_	CH_4_	NH_3_	Acetic Acid	Propionic Acid	*n*-Butyric Acid	IVDMD	IVOMD
WBC	1	0.85	−0.27	−0.11	0.09	0.12	0.28	−0.34	0.08	−0.04	−0.27	−0.35	−0.29
WHC		1	0.05	−0.43	0.48	0.49	0.39	−0.25	0.42	0.33	0.02	−0.03	0.05
SC			1	−0.68	0.52	0.50	−0.08	−0.30	0.47	0.64	0.16	0.45	0.48
pH				1	−0.85	−0.88	−0.28	−0.06	−0.78	−0.82	−0.51	−0.47	−0.54
GP_36_					1	0.99	0.30	0.15	0.78	0.77	0.57	0.56	0.67
GP_48_						1	0.35	0.22	0.77	0.76	0.58	0.56	0.67
CH_4_							1	0.38	0.58	0.38	0.36	0.11	0.08
NH_3_								1	0.26	0.20	0.68	0.33	0.29
Acetic acid									1	0.88	0.69	0.52	0.58
Propionic acid										1	0.71	0.54	0.61
*n*-Butyric acid											1	0.57	0.58
IVDMD												1	0.96
IVOMD													1

GP_36_: gas production measured after 36 h of incubation; GP_48_: gas production measured after 48 h of incubation; IVDMD: in vitro dry matter digestibility; IVOMD: in vitro organic matter digestibility; SC: swelling capacity; WBC: water binding capacity; WHC: water holding capacity. Significant correlations are highlighted (*p* < 0.05).

## Data Availability

The data presented in this study are available on request from the corresponding author.
